# Editorial: Current Issues of Trauma and Technology Use

**DOI:** 10.3389/fpsyg.2022.935044

**Published:** 2022-06-01

**Authors:** Xiaoyu Zhuang, Miguel Ribeiro Ramos, Xue Yang

**Affiliations:** ^1^Sociology Research Center, School of Humanities, Jinan University, Guangzhou, China; ^2^Department of Social Policy, Sociology, and Criminology, University of Birmingham, Birmingham, United Kingdom; ^3^School of Public Health and Primary Care, The Chinese University of Hong Kong, Hong Kong, Hong Kong SAR, China

**Keywords:** digital use, traumatic experience, mental health, behavioral health, addiction

Trauma (e.g., social and environmental changes, collective traumatic events, stressful daily life events) may have short-term and long-term influences on individual behaviors and wellbeing. With the popularity of computers and smartphones, technology use plays an increasingly important role in coping with trauma and related stress. This Research Topic curated a collection of papers that are representative of current trends and advances in exploring the complex role of technology use in traumatic events.

First, Yang et al. adopted a population-based random community sample to explore the epidemiology of Internet gaming disorder (IGD) when strict COVID-19 mitigation measures were implemented in Hong Kong. The weighted prevalence of IGD was found to be 9.7%, higher than that of pre-COVID-19 research. This finding echoed other studies mostly conducted among young people during the COVID-19 pandemic. This study was also the first to empirically examine the role of post-traumatic stress resulting from repeated exposures to negative stressful events stemming from the worldwide pandemic in influencing problem technology use (PTU). Moreover, based on the Conservation of Resources Theory, they unveiled inconsistent mediating effects of two psychological statuses, boredom and loneliness, in explaining the complex mechanisms of the relationship between social media use and social media addiction.

Second, Guggisberg et al. reported a systematic review on the critical indicators of post-traumatic growth (PTG) among female victim/survivors of sexual violence. Previous studies only documented the devastating mental and physical health consequences of sexual victimization. Recent studies started to explore whether and how the female victim of sexual violence may achieve recovery and resilience indicated by PTG. The authors highlighted common themes that may contribute to PTG, including meaning-making, cognitive appraisal, having control and decision-making abilities, altruistic actions and activism, and helping other victims. Moreover, they further abstracted the themes into two superordinate topics, which were defined as “relationship to self” and “relationship to others.” They discussed how technology use may facilitate women to regain meaning by speaking out in social media and forming recovery or peer support groups in the digital community.

In the third paper, a research team from Germany (Leo et al.) reported a 12-month longitudinal study that examined the causal effects of clinically-diagnosed depression and social anxiety on Internet use disorder symptoms in children and adolescents. Positive effects were demonstrated. This study is the first to examine whether and how clinical samples may develop problematic internet use (PIU). Clinical samples may share common and different traits, states, and motives with non-clinical samples which may affect the development and manifestation of PIU. For example, maladaptive emotion regulation and maladaptive reward processing (i.e., reward deprivation) may be pivotal mechanisms explaining how internalizing disorders such as depression and anxiety might predict externalizing problems such as PIU (Leo et al.).

The last paper coined by Li et al. from Hubei, China, reported a dual mediation model that explained how peer victimization predicted problematic online game use among Chinese adolescents. Anchored in the ecological system theory, the study demonstrated that those traumatic experiences might impact adolescents' interpersonal and school systems by engendering deviant peer affiliation and jeopardizing school connectedness, which in turn resulted in PTU. The findings imply that it is important to strengthen school connectedness and improve peer relationships to reduce the possibility of problematic online game use among Chinese adolescents.

## Future Research Directions

First, given the promising conducive role of using social media to regain meaning and sense of control for stigmatized groups who have experienced trauma such as female victims of sexual violence, we suggest that future studies may further explore how social media may facilitate PTG in different stigmatized groups and whether these groups are more likely to develop PTU since they may be likely to rely on social media to cope with their stress, stigma, and negative emotions. Second, efforts have been made to propose and examine critical psychosocial mechanisms at intrapersonal (e.g., psychological statues, maladaptive emotion regulation) and interpersonal levels (e.g., peer affiliation, school connections) to account for the development and maintenance of PTU. Mechanisms should be explored to enrich the knowledge base of the etiology and pathology of PTU in the context of trauma. Finally, we suggest that more longitudinal studies in different age and sex groups should be conducted to comprehensively understand the complex roles of technology use in trauma. For young females, their IGD may be a hidden and understudied problem. The “gender gaps” in gaming participation and IGD prevalence may be further narrowed due to the collective traumatic events such as the COVID-19 isolation. Whether PTU would increase during trauma and remain or drop after trauma in children vs. adults should be monitored. Such surveillance would help to understand the nature of PTU (e.g., stability over time, incidence, and remission rate), estimate healthcare service needs, and design health promotion strategies for early detection and treatment.

## Author Contributions

XZ and XY were responsible for paper drafting. MR was responsible for paper editing. All authors contributed to the article and approved the submitted version.

## Conflict of Interest

The authors declare that the research was conducted in the absence of any commercial or financial relationships that could be construed as a potential conflict of interest.

## Publisher's Note

All claims expressed in this article are solely those of the authors and do not necessarily represent those of their affiliated organizations, or those of the publisher, the editors and the reviewers. Any product that may be evaluated in this article, or claim that may be made by its manufacturer, is not guaranteed or endorsed by the publisher.

